# Probe and Sensors Development for Level Measurement of Fats, Oils and Grease in Grease Boxes

**DOI:** 10.3390/s16091517

**Published:** 2016-09-16

**Authors:** José Faria, André Sousa, Arsénio Reis, Vitor Filipe, João Barroso

**Affiliations:** 1 ECT, Universidade de Trás-os-Montes e Alto Douro, Vila Real 5000, Portugal; jfaria@utad.pt; 2 INESC TEC and Universidade de Trás-os-Montes e Alto Douro, Vila Real 5000, Portugal; andresousa@utad.pt (A.S.); vfilipe@utad.pt (V.F.); jbarroso@utad.pt (J.B.)

**Keywords:** sensors, probes, FOG detection, grease box, capacitance sensor, conductivity sensor

## Abstract

The wide spread of food outlets has become an environmental and sanitation infrastructure problem, due to Fats, Oils and Grease (FOG). A grease box is used at the industrials facilities to collect the FOG, in a specific time window, while its quality is good for recycling (e.g., biodiesel) and it is economically valuable. After this period, it will be disposed at a cost. For the proper management of the grease boxes, it is necessary to know the quantity of FOG inside the boxes, which is a major problem, as the boxes are sealed and permanently filled with water. The lack of homogeneity of the FOG renders it not detectable by current probes for level detection in liquids. In this article, the design, development and testing of a set of probes for FOG level measurement, based on the principles used in sensors for the detection of liquids inside containers, is described. The most suitable probe, based on the capacitance principle, together with the necessary hardware and software modules for data acquisition and transmission, was developed and tested. After the development phase, the probe was integrated on a metropolitan system for FOG collection and grease box management in partnership with a grease box management company.

## 1. Introduction

The food industry is a major water pollutant, dumping Fats, Oils and Grease (FOG) [1] in the sewerage system. The residues from industrial kitchens cause problems, such as the reduction of oxygen available in the water and the formation of layers of fat on the water surface, dramatically affecting the ecosystems in which water plays an important role [2]. Due to this problem, death of aquatic and terrestrial animals has been recorded [3]. Besides the environmental problem, there is also a negative effect on the sewerage infrastructures as well. Over time, these infrastructures get clogged with fat, which solidifies at lower temperatures, reducing the water flow, corroding the sewage pipes, and thus increasing the risk of sewage spillage [4,5,6,7].

To deal with this problem, water treatment plants have specific processes to remove fat and mud from water, mainly through the usage of enzymatic treatments that break down the fat molecular chains. Although these treatments may neutralize the fat and acidity, allowing the separation of the waste from the water, the recovered FOG is actually a mud residue unusable or recyclable. To prevent the negative consequences of FOG to the sewerage system, as well as the necessity for specific treatments at the water treatment plants, governments and regulators have created laws and regulations in order to have some sort of pretreatment, at the industrial facilities, before dumping the wastewater into the sewerage systems. One the most important components of the pretreatment infrastructure is the grease box, which contains a grease trap and separates the fat (oil and grease) from the wastewater, trapping the oil and grease in the box and delivering the water to the sewerage system. Once trapped in the grease box, oil and grease must be collected before filling up the box and spill to the sewerage system. Timely collection is critical because, while staying in the grease box, oil and grease will be in contact with other waste, such as cooked rice grains and small chips of cooked meat, which will cause the oil and grease to become more acidic over time. If oil and grease collection occurs soon after the filling of the grease box, then most of the oil and grease will have good enough quality to be recycled, e.g., for biodiesel, and will have economic value as a revenue [8,9]. Otherwise, the acidity level will rise to a point where the oil and grease will not be recyclable and will have to be collected and disposed at a cost as a residue [10].

**Figure 1:**
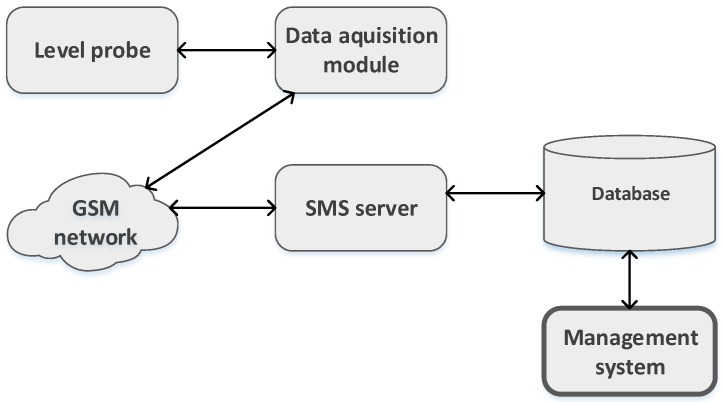
Grease boxes monitoring and management system.

This article presents the work of developing a system to monitor the grease boxes and alert for the correct time of collecting the oil and grease trapped in the box. For this purpose, we research the most adequate sensors for measuring the grease box fill levels, having tested and developed special purpose sensors, based on the principle of the different electric conductivity, density and capacitance of the water and grease filling a box. Together with the sensors, a module of data acquisition and communication was also developed. The grease boxes, equipped with these sensors and the data acquisition and communication modules are able to send messages, alerting for the best time to collect the grease, in order to have a product, at least partially recyclable. A central server, for receiving the grease box messages, management and configuration of the grease boxes was also developed. Figure 1 represents a general view of the complete monitor and management system.

This system was implemented, as a prototype, in partnership with a company dedicated to the collection of used cooking FOG and grease boxes cleaning and management.

## 2. Theoretical Basis and Methods

In this section, we present the current scenario regarding grease boxes design and the suitability of the methods currently available to detect and measure the level of FOG inside the boxes.

### 2.1. Grease Boxes 

**Figure 2:**
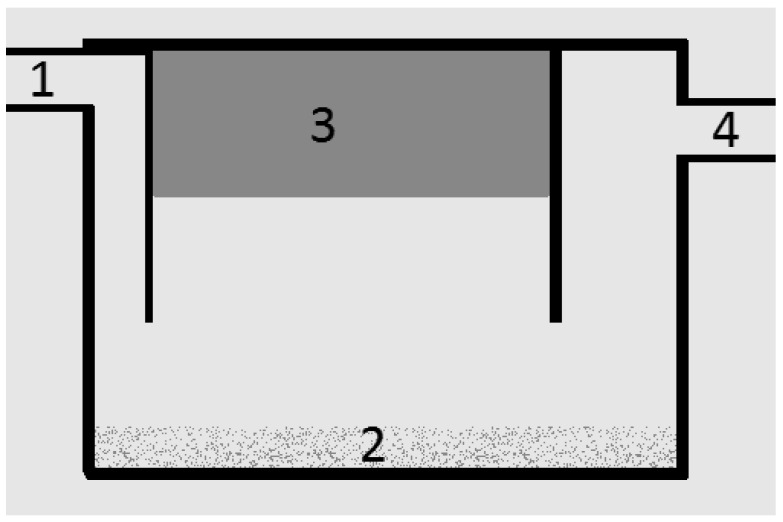
The grease box composition: 1, wastewater entrance; 2, solid residues; 3, fats; and 4, exit to sewage.

Grease boxes are designed according to Figure 2 and operate under two simple principles: water and FOG do not mix; and FOG is less dense than water [11,12]. On installation or cleaning, the grease box is filled with water. When oils, fats and other residues are dumped in the kitchen sink, heavy materials, e.g., fine grain sand, will accumulate in the bottom of the grease trap, while FOG will float and accumulate on the water surface at the top. In the central level of the trap, there will be water and residues with the same density as water, which will be released to the sewerage system. In Portugal, there is a legal limit of 15 mg of FOG per liter of water so it can be legally discharged in to the sewerage system [13].

FOG recollection and grease box cleaning is done by sucking the entire content of the box into a transport vessel and storage in a treatment plant, after which it will go through a treatment process, including separation of FOG, water and heavy materials.

Probes for measuring the level of a liquid in a container can be divided in two categories according to the type of sensors used:

- Continuous measurement sensors that are able to provide the actual level of the liquid; and
- Presence measurement sensors, which can only provide a confirmation that the liquid has actually reached the level where the sensor is installed [14].

In this work, we studied both types of probes and sensors, their suitability, as well as the best approach for level detection in the specific case of a grease box. 

### 2.2. Detecting the Level of FOG Inside A Container

The detection of the FOG level inside the grease box is the major problem regarding the implementation of a monitoring and management system. Five sensor types are studied in order evaluate their usage in building a probe for FOG level detection. 

#### 2.2.1. Conductivity Sensor

Conductivity sensors work based on the fact that water conducts electricity and, among other applications, are commonly used for getting a rough estimation of the water level in water wells. For this purpose, three metal rods are installed at different levels in the well and connected to a level relay (electric relay) that will open and close, based on the water electric conductivity, and thus commanding power equipment, such as pumps, according to the water level. The same principle can be used for grease boxes, considering that vegetable oil and animal fat, used in cooking, do not conduct electricity, and that the grease box is filled with water up to a level and with fat and oil above that level. 

These level relays are used in large grease boxes, working under the same principle, in order to activate an alert device, such as a bell or a light, when the box is ready for cleaning. It is a simple mechanism but requires some care with the probes (metal rods) as the accumulation of FOG affects the system sensibility.

#### 2.2.2. Level Buoy with Magnetic Sensor

Level buoys are a common, inexpensive and efficient solution for measuring the level of a liquid in a container, especially when conductivity sensors cannot be used due to poor conductivity of the liquid or fire hazard. The buoy should be less dense than the liquid, so it floats at the top. Due to the moving parts, there is a high mechanical complexity associated with this sensors as well as high construction costs.

As the grease box is completely filled with water and FOG at all time, to use this method to measure the level of FOG accumulation, the buoy should be more dense then the FOG and less dense than the water. That way it would float in between the water and the FOG. A magnetic sensor, electronic [15] or mechanic [16], would be mounted in the buoy axis or outside the box, at a desired level and connect to a level alert system.

#### 2.2.3. Optical Sensor

The optical sensor works by measuring the quantity of light inside a probe, which is generally built as a transparent cone or prism with a well-known refraction index, usually 1.5. Inside the probe a light emitter and a light receptor are installed [17]. When used to detect water, two scenarios can occur: the sensor is out in the air (refraction index 1) and a certain quantity of the emitted light is reflected back to the receptor; the probe’s tip is submerged in water causing the angle of reflection inside the probe to change, as well as the quantity of light reaching the receptor, making it possible to detect the immersion of the probe’s tip in the water.

The angle of the cone or prism is set according to the refraction index of the liquid to be measured, making it possible to build a probe capable of detecting the difference between the refraction index of the water (about 1.333) and the index of the oil (generally superior to 1.333) [18]. It is a simple and inexpensive sensor that may not be usable in the grease boxes context due to the strong possibility of formation of a grease film on its surface, altering the reflective properties and the detection capabilities of the sensor.

#### 2.2.4. Density Sensor with a Load Cell

This type of sensor is similar to the level buoy sensor, with the difference being in the detection, which in this case is accomplished with a load cell connected to the buoy by means of a rod. The load cell measures the force on the buoy and, because water is denser than FOG, the pressure on the load cell should be bigger when the box is full of water, decreasing as the water is replaced by FOG inside the box. This sensor is complex and expensive, considering the mechanical parts as well as the electronics needed to effectively use the load cell. 

#### 2.2.5. Capacitance Sensor

The capacitance sensor uses the electric capacitor working principle in order to measure the level of a liquid in a container [19]. The capacitance between two plates can be formulated as $∁=\frac{kA}{d}$, in which “*C*” is the capacitance, “*k*” is the dielectric constant of the material between the plates, “*A*” is the area of the plates, and “*d*” is the distance between the plates. When the container is an electrical conductor, a sensor element is inserted inside the container, which measures the capacitance between the container wall and the sensor element. As the quantity of liquid changes inside the container, the capacitance also changes. The difference in capacitance can be used to measure the level of the liquid. In the case the container is an electrical insulator, a second sensor element must be inserted and the measurements are taken between the two elements. The most common capacitance sensors are continuous measurement type with an analog output of 4 mA to 20 mA, which falls in the standard for industrial automation [20], and sensors with a RS-232 or RS-485 digital output [21].

Although it is an efficient type of sensor, it is also very expensive [22]. There are simpler and less costly versions of this sensor that just measure changes on a capacitance basis on the effect of touching one of the capacitor plates [23]. Good examples are capacitance touch screens and buttons. 

## 3. FOG Level Detection: Implementation and Results

Based on the previously studied sensors, three probes, based on three different sensors (conductivity, density and capacitance) with the specific purpose of detecting the FOG level on the grease trap part of the grease boxes, were developed. The main objective was to test and select the best probe to use in the grease box monitoring and management system. The development and test was done in two phases: a first phase in laboratory and a second phase in a restaurant in partnership with the FOG management company.

### 3.1. The Laboratory Development Environment and Test Bed

**Figure 3:**
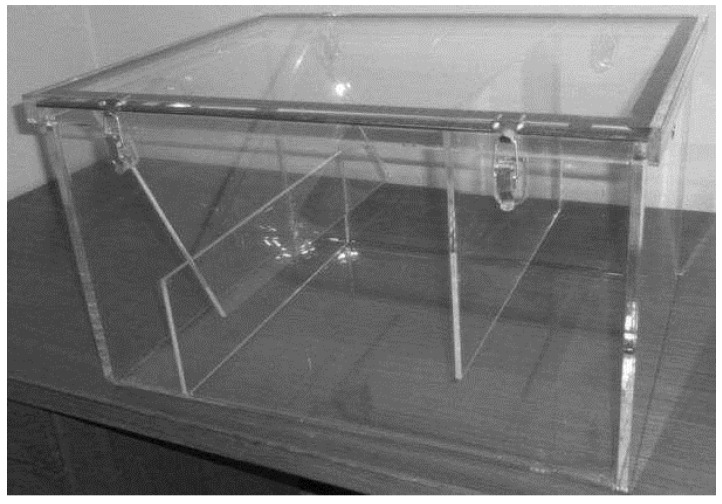
Prototype test boxes.

As a test bed environment, a transparent grease box, allowing a fast visual assessment of the FOG level as well as a good control of the field tests, was built. For the preliminary tests, mainly to assure the validation of the readings and the prototype water tightness, a small dimensions box was built, as shown in Figure 3.

The results were visually validated, which is very important because FOG varies in composition and form.

**Figure 4:**
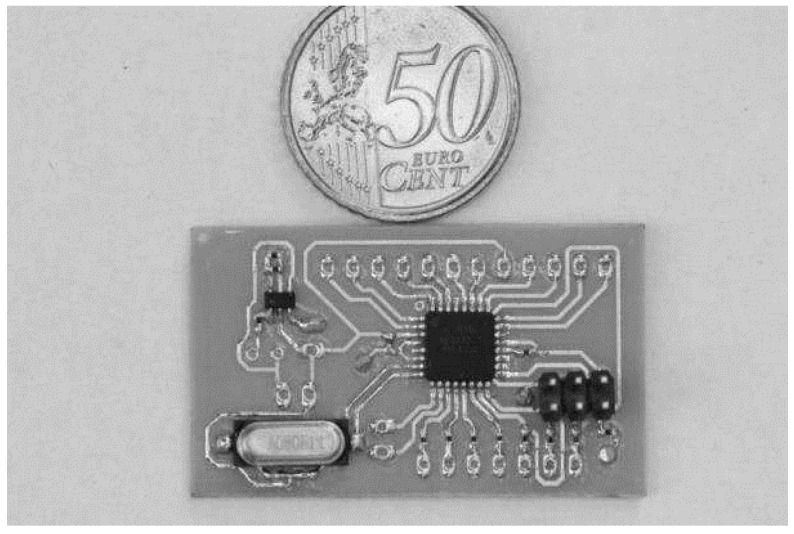
The data acquisition module.

A data acquisition system was also developed and built, as shown in Figure 4. In the laboratory tests, the sensors readings were transmitted using a serial port (RS232) and stored in a computer. 

**Figure 5:**
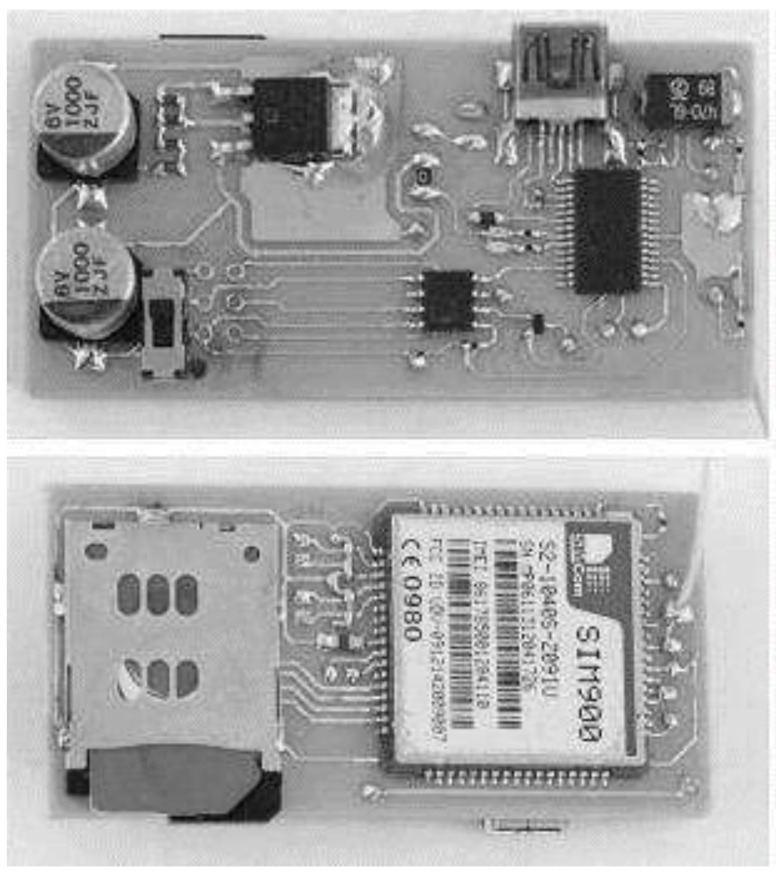
GSM SMS messaging module.

In the field, the readings were sent by Short Message Service (SMS), to a Global System for Mobile Communications (GSM) gateway connected to the central system by means of a Universal Serial Bus (USB) port. Figure 5 illustrates the GSM messaging module [24,25].

### 3.2. Conductivity Probe

Because the conductivity principle is simple and can be used to build fairly inexpensive probes, the first probe was based on this principle. In the prototype, the acquisition module receives readings from seven metallic probes, whose conductivity varies according to the environment of water or FOG. The probes were located vertically on the container wall, with the probe in the bottom acting as reference (water) and the other six individually connected to an ADC input pin of the microcontroller ATmega328 (Atmel Corporation , San Jose, CA, USA) [26]. 

**Figure 6:**
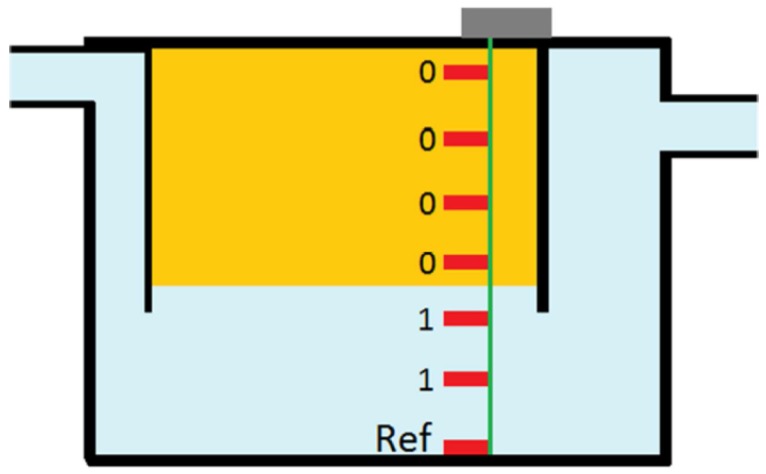
Conductivity probes (in red) in a grease box.

When the box is empty of FOG (full of water), all pins are connected to the 5 V reference and have the 1023 value (maximum value for the 10 bits ADC). As the FOG level raises, the probes will become individually isolated, and have a 0 value, due to lack of electric contact with the bottom reference probe. Figure 6 illustrates this working principle.

In the laboratory tests, the conductivity probes were effective in detecting the vegetable oil that was added to the water content of the grease box. In the field tests, conducted in a partner restaurant, the system was unable to obtain proper readings as all pins would present readings above 1000, even with the probes apparently covered with FOG. After a careful analysis of the FOG trap, we concluded that in the small boxes of 50 L, the FOG does not accumulate in a uniform way, as with the oil in the lab tests, but instead, the kitchen fat and oil residues tend to form blobs of various sizes with water between, thus not allowing for true electric isolation of the probes.

### 3.3. Density Probe

**Figure 7:**
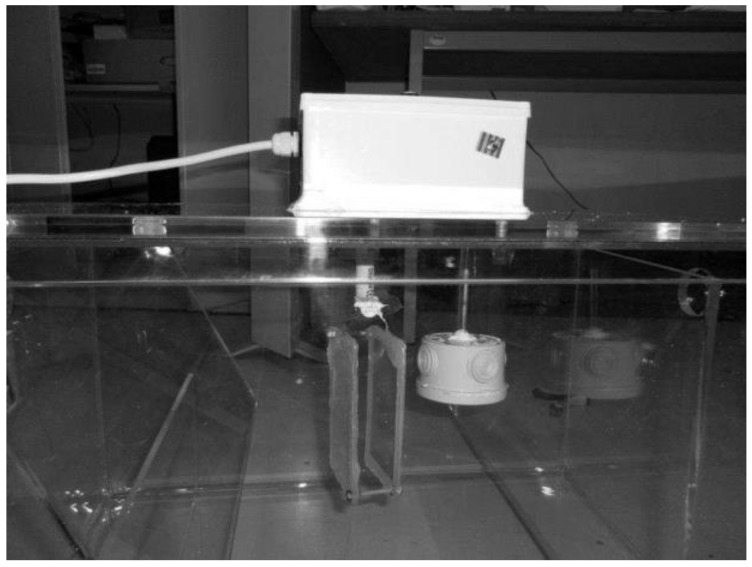
Density measurement with a buoy and a load cell.

The prototype of the density probe (Figure 7) was implemented using a buoy with a vertical rod connected to a load cell. The buoy detects density changes in the content of the grease box, activating the load cell thought the pressure force of the vertical rod. The signal from the load cell is then amplified by an instrumentation amplifier (AD623, Analog Devices, Inc., Norwood, MA, USA) [27] and converted in to a digital signal by an ADC.

The prototype was later tested in a restaurant, in a real scenario. The microcontroller of the acquisition module was programed to run daily readings and send them by SMS using the integrated GSM module. During the tests, we concluded that the sensor did not have enough sensitivity to differentiate between the density of water and the density of FOG. The lack of sensitivity is most likely due to the mechanical parts. In addition, this prototype uses expensive components, such as an instrumentation amplifier, and for those reasons this method of measurement was unsuccessful.

### 3.4. Capacitance Probe

**Figure 8:**
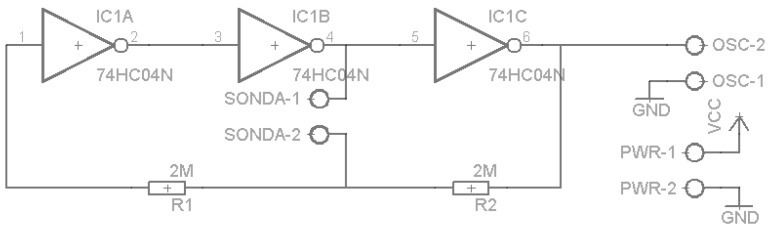
Capacitive probe’s oscillator circuit.

The capacitance probe was implemented as a pair of parallel plates, forming a capacitor, connected to a simple oscillator, as described in Figure 7 and Figure 8.

The oscillator output frequency depends of the values of the two resistances (R1 and R2) and the capacitor. It can be estimated by the formula $f=\frac{0.559}{R*C}$ [28]. Under the capacitance principle, a change in the content of the grease box will induce a change in the capacitance of the capacitor formed by the pair of plates and a change in the output frequency of the oscillator.

**Figure 9:**
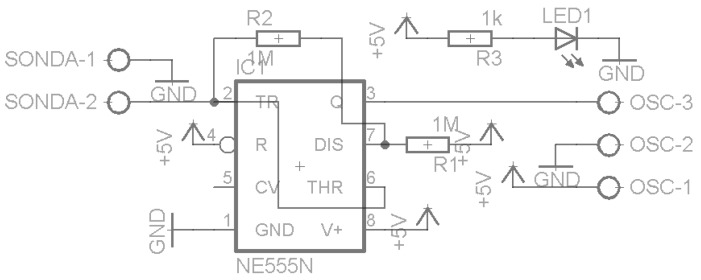
Circuit oscillator of the second prototype.

In the laboratory tests, it was verified that the changes in capacitance was very small and easily contaminated by the high capacitance of the cable (30 cm cable) connecting the plates to the oscillator. A new prototype was built with two elements made of aluminum tube. The oscillator was miniaturized and the circuit board was designed to be placed inside the reference element (earth) without cables, as shown in Figure 9.

**Figure 10:**
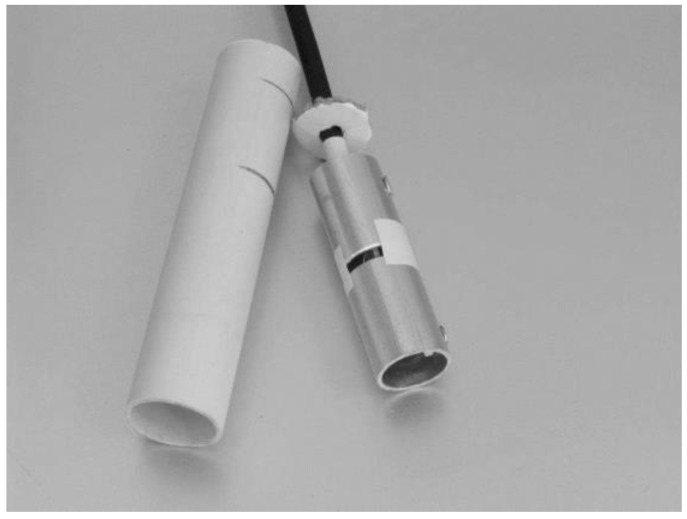
Capacitance probe with the plastic container disassembled.

**Figure 11:**
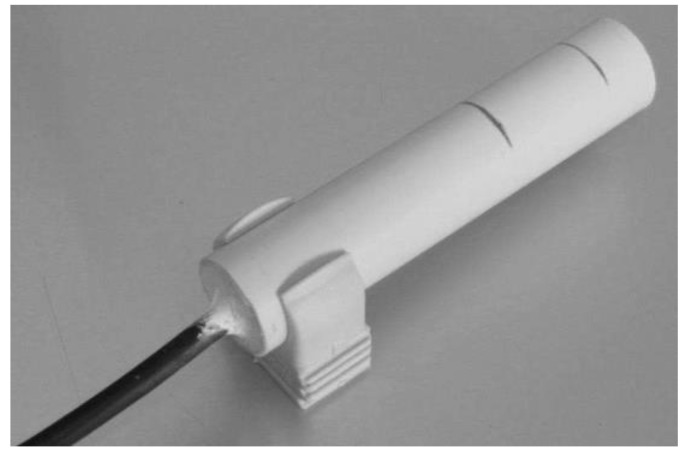
Capacitance probe fully assembled.

Figure 9 illustrates the oscillator design of the second prototype, based on a timer chip. To protect the aluminum elements, a plastic tube glued and sealed around the elements was used, as shown in Figure 10 and Figure 11.

The oscillator is based on a CMOS timer chip [29] in order to reduce the energy consumption as well to facilitate the configuration of the output signal. The capacitance of the probe and the wires can be calculated as a function of the frequency in the oscillator’s output as $C=\frac{1.38}{2100000*f}$, in which *C* is the probe’s capacitance and f is the oscillator’s frequency.

### 3.5. Testing the Capacitance Probe

**Figure 12:**
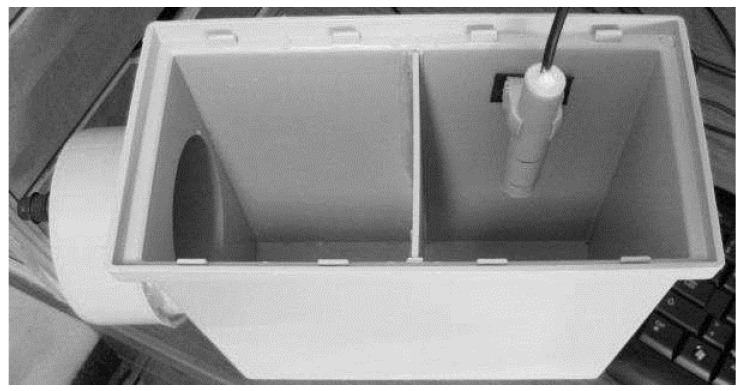
Test box used for in lab prototype testing.

The laboratory tests were conducted using the box in Figure 12 and by fully submerging the probe in water and then adding vegetable oil to the probe’s compartment in order to have the water progressively replaced by oil. For these specific tests, we did not use transparent boxes, but we knew the box capacity as well as the amount of oil or FOG that was inserted into the box. Because the box is always full, by knowing how much FOG was inserted, we also knew how much water was in the box and their respective levels.

**Figure 13:**
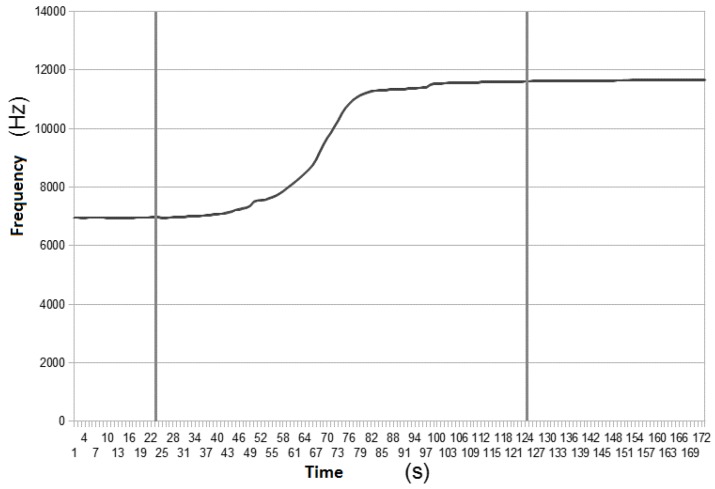
Laboratory tests results.

In Figure 13, the samples retrieved at a sampling rate of 1 Hz as the oil was added to the box are displayed. 

The first sample shows the oscillation frequency with the box full of water and, from the previous cited function, it is possible to estimate the probe’s capacitance in water as 94 pF. After the second sample, a liter of vegetable oil was added, filling the box retention capacity, at a constant rate of 0.5 L per minute. At the 23º sample, the oil reached the probe, after which the oscillation frequency started to increase. At the 125ª sample, the probe was completely immersed in oil and although more oil was added to the box in the following samples, the frequency value did not change. The probe’s capacitance when immersed in oil is about 56 pF, presenting a 38 pF difference to when it is immersed in water.

**Figure 14:**
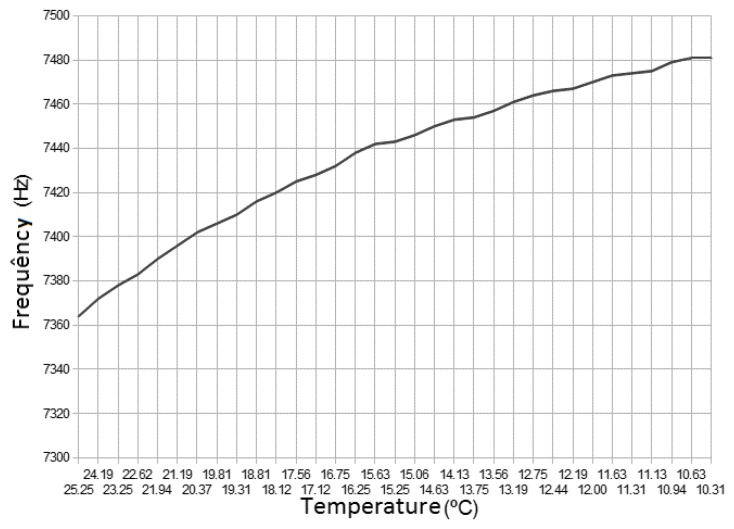
Frequency variation.

To verify the effect of probe’s temperature variation on the output frequency, the previous setup with the water heated to 25 °C and progressively cooled down to 10 °C was used. Figure 14 shows that the temperature affects the oscillation frequency on a predictable way. The frequency increase while the temperature decreases is a cumulative effect due to the changing in the dielectric constant of the water and the variation of the component’s values.

**Figure 15:**
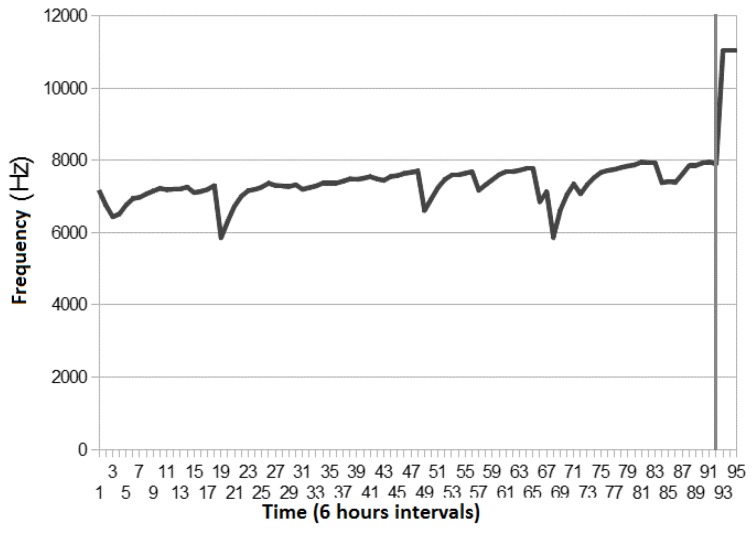
Tests results in a real production environment.

After the laboratory tests, the probe was tested on the transparent box, installed on real environment of a partner restaurant. The data acquisition module was programmed to acquire and transmit frequency readings in 6 hours periods using SMS messages. Figure 15 represents the received values. The sampling frequency of six hours was established based on the regular working schedule of a restaurant.

The vertical line on the graphic in Figure 15 marks the moment when the probe was enclosed in oil, after which the readings values are clearly superiors. The down peaks in the graph are caused by the usage of hot water that heats the entire box and causes the oscillation frequency to drop as the temperature rises.

**Figure 16:**
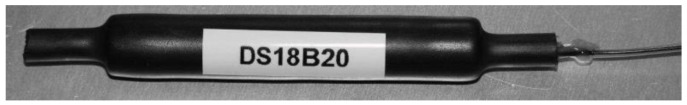
Digital temperature sensor with a waterproof container.

**Figure 17:**
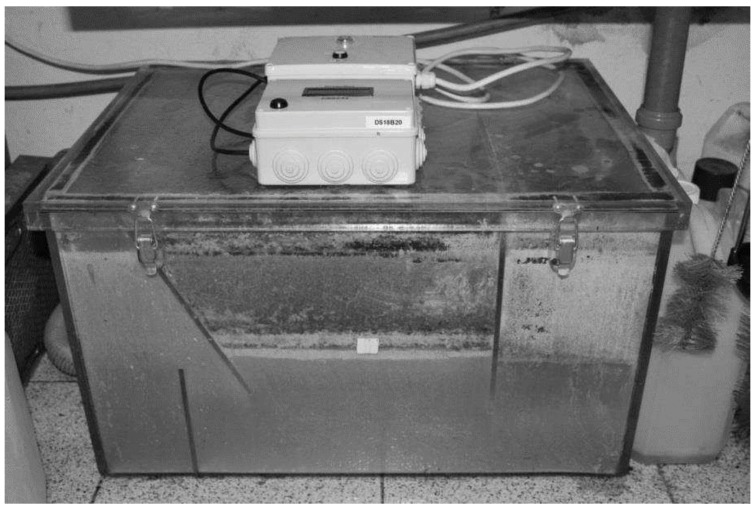
Transparent grease box for temperature monitoring.

For a better understanding of the factors affecting the results, a temperature sensor was added to the probes, as shown in Figure 16 and Figure 17, based on the DS18B20 chip (Maxim Integrated, San Jose, CA, USA) [30], which was connected to an acquisition module with an LCD for local monitoring of the temperature inside the box. 

**Figure 18:**
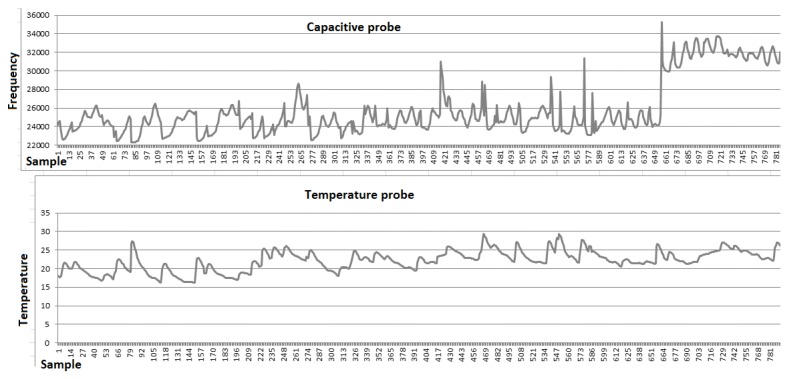
Temperature influence in a real production test.

Figure 18 represents one of the tests, in which, even with some noise, the effect of the temperature in the frequency reading acquired from the capacitance probe is visible.

**Figure 19:**
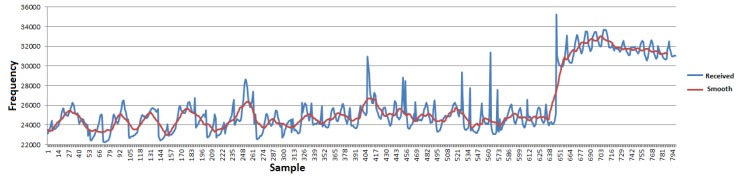
Smoothing the capacitive probe signal.

To eliminate the noise in the signal acquired from the capacitance probe, a filter in the reception program, based on a smoothing function, was implemented. Figure 19 shows the graphs of the acquired signal before and after applying the smoothing function.

The analysis of the graphs in Figure 19 lead to the conclusion that, using the filtered data, values exceeding 29 kHz confidently indicate the presence of FOG on the probe. Applying the same criteria to the raw data, there are two false positives, on sample 407 and sample 563, caused by the noise. An alternative way to filter false positives without the use of averages or other smoothing functions, which require historical data, is to use a certain number of consecutive readings above a predefined value before triggering the alarm of the presence of FOG.

Several probes were built using metallic elements aluminum, copper and steel; however, more important than the metal, is the size of the elements, which influences the values of the readings. Bigger elements yield higher capacitances and lower oscillation frequency.

### 3.6. Production Capacitive Probe

The full production capacitive probe was built and integrated in a monitoring system with a similar design to those described in the previous section for the prototype. The format and the materials were chosen in order to maximize performance, ease of construction and low cost.

For the elements, a stainless steel tube with 22 mm in diameter, cut with a 2 cm length was used. Elements with a bigger length would have bigger detection areas, making it hard to detect, with precision, if the FOG has reached a predefined level. Elements with a smaller length would have an oscillation frequency too high and incompatible with a low clock frequency microcontroller. The probe’s container was built with plastic tub 25 mm in diameter and 10 cm in length.

**Figure 20:**
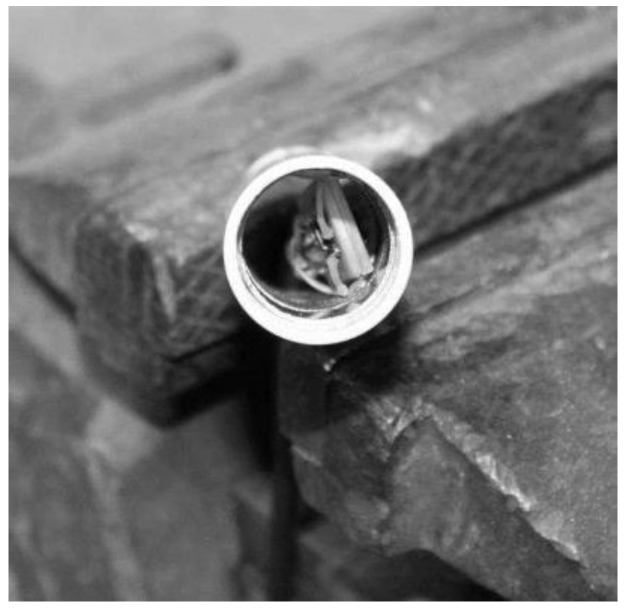
The probe before soldering and sealing.

**Figure 21:**
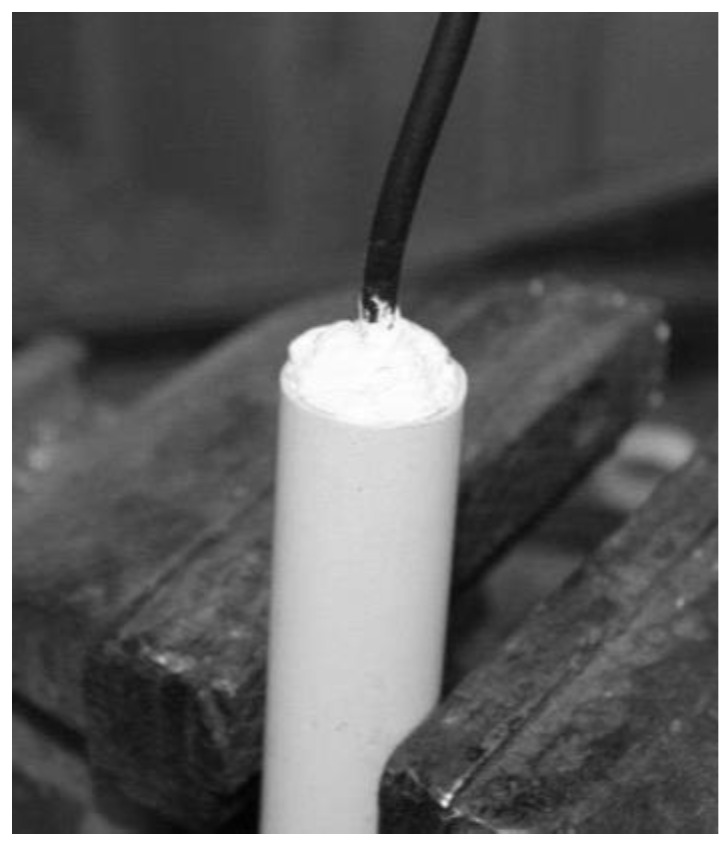
The final probe.

A connecting electric wire is soldered to each element and the container is glued and waterproof sealed. In Figure 20 and Figure 21, the probe before soldering and sealing and fully finished is shown. When constructing the probes, it is very important to have quality control and assure the compliance with the specifications, as small differences in the size of the elements will lead to big differences in the output frequency.

**Figure 22:**
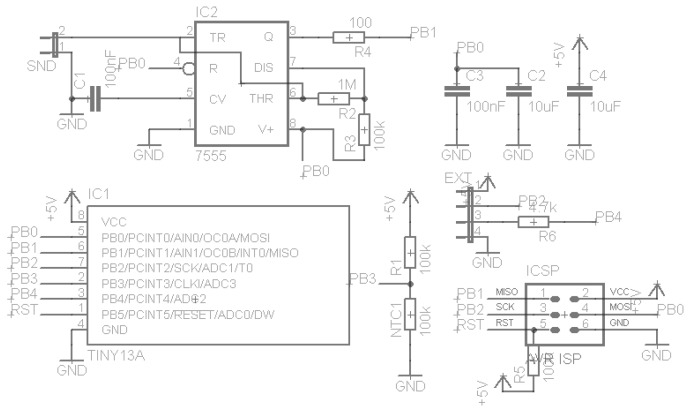
The probe with an integrated ATiny13a microcontroller.

In order to manage the probe’s readings, an ATiny13a microcontroller (Atmel Corporation, San Jose, CA, USA) [31], as represented in Figure 22, was added.

The microcontroller was programmed to supply energy to the oscillator using the PB0 pin, wait until the oscillator stabilizes, and count the number of pulses in the PB1 pin during 100 ms, after which the oscillation frequency with a 10 Hz resolution is obtained. Then, the voltage in pin PB3 using the ADC, connected to a thermistor in order to have an estimative of the probe’s internal temperature, is read. The values are then converted to text and sent to the acquisition module by the pin PB2, using a software implementation of a UART port, as the microcontroller, does not have a UART hardware module. The microcontroller was chosen for its small size and low cost and was programmed using the C language.

For the tests process, an acquisition module with storage capacity, in order to store the reading and automate data collection in the laboratory was built. In Table 1, the probe’s readings at several temperatures when in contact with the air are presented.

Using the results in Table 1, the following compensation formula in order to compensate the temperature variation was designed: *C* = *f* + ((CAL − ADC) × 25), in which “*C*” is the compensated value, “*f*” is the read frequency, “CAL” is the calibration value, and “ADC” is the value read from the ADC. The probe is calibrated at 18 °C, with an ADC value of 608, by applying the compensation formula, *C* = *f* + ((608 − ADC) × 25), and the results are shown in Table 2. Compensated results from readings are shown in Table 1. Table 2 results are obtained and it can be verified that the compensation formula provides a proper approximation to the noise margin, considering that in similar conditions, due to noise, the output frequency can vary by about 1 kHz.

**Figure 23:**
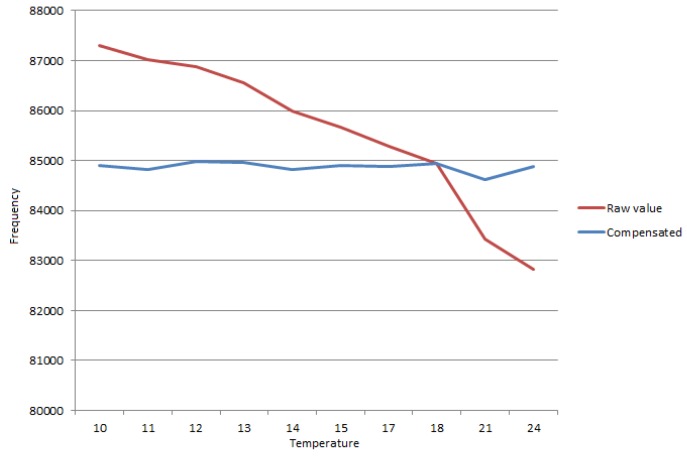
Raw oscillation frequency reading and compensated frequency reading.

In Figure 23 and Table 2, the effect of the compensation formula on removing the effect of the temperature in the oscillation frequency is shown.

**Figure 24:**
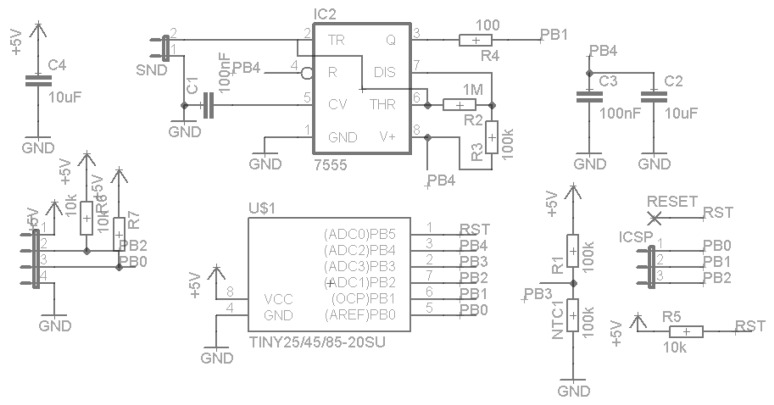
The probe with the ATiny25 microcontroller.

The firmware built for the tests used most of the available memory space for program code in the microcontroller (1 kB). To implement the additional functionalities and to simplify the communication process between the probes and the acquisition module, the ATiny13a microcontroller was replaced by an ATiny25 (Atmel Corporation, San Jose, CA, USA) [32], as shown in Figure 24.

The ATiny25 microcontroller (Atmel Corporation, San Jose, CA, USA) has a bigger code storage capacity (2 kB) and a Universal Serial Interface (USI) hardware module, capable of using several protocols [32,33]. The selected protocol was I2C, which uses two wires: a clock line and a data line, in half-duplex [34]. 

Commonly known as Two Wire Interface, the I2C protocol supports several master and slave in one single bus. It was created by Philips and specially designed for low speed communication between electronic devices. Intel, in 1995, designed the SMBus, based on I2C, but with more restricted tolerances, allowing it to be used with I2C devices in SMBus, with small adjustments [35]. In the original specification, the I2C protocol had a maximum clock speed of 100 kHz, which is now 5 MHz in version 4, released in 2012 [36]. The clock speed, defined by the master device, can have an arbitrary value, no higher than the maximum limit of the slowest slave device in the bus. In the current case of the capacitance probe, the speed is directly proportional to the clock speed of the microcontroller, i.e., the higher the clock frequency of the internal oscillator, the higher the maximum speed of the USI module and also the higher the power usage. When using several master devices on the same bus, there is the possibility of two masters trying to initiate the communication at the same time, so I2C uses the Arbitration method to avoid collisions. For the addresses, I2C uses seven bits and reserves 16 addresses, thus allowing the usage of 112 slave devices with each master device [37]. I2C is a simple protocol and is used worldwide as an industry standard [34].

The default address for the probe is 0 × 26 and can be changed by the master in a scenario with several probes on the same bus. The commands implemented in the probe’s firmware are presented in Table 3. Command to configure and operate the probe Table 3.

**Figure 25:**
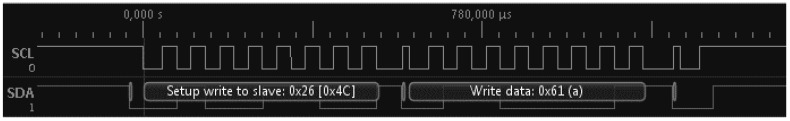
Clock line and data line of a sample acquisition request to a probe with the 0 × 26 address.

In Figure 25, the communication of a reading request of a sample, as captured by a logic analyzer is represented [38]. The Probes address, 0 × 26, in the beginning of the message will inform the other devices on this same bus to ignore the message.

**Figure 26:**
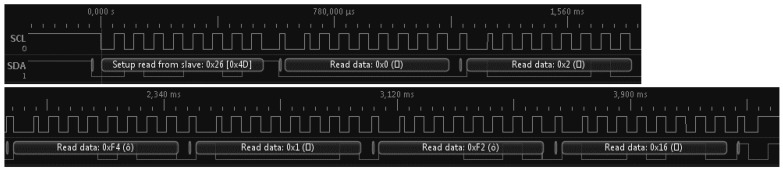
Probe’s reply to the sample acquisition request.

After this request, the acquisition module will wait the necessary time for the probe to have the data ready to send, as in the I2C protocol only the master can change the clock line, so the slave device has no means of signaling when the sample is ready. After the waiting period, the master requests an answer to the probe, as represented in Figure 26. This request is initiated with the probe’s address, so only the probe with the 0 × 26 address has authorization to send data, after which six bytes are received with the meaning (Table 4).

In the probe, the frequency is read to a four-byte variable, of which only three bytes are effectively used, and the temperature, as read by the ADC, is stored in a two-byte variable. Because I2C can only send eight bits simultaneously, so it is necessary to divide the variables into eight-bit blocks. When the “usiTwinTransmitByte (char c)” is called, the byte sent as parameter is stored in a buffer and sent as soon as the master request a data communication. The variable “soma” is used as a checksum, in order to provide a way to validate the communication quality, after which the received data are processed in the acquisition module. 

The probe’s serial number, address and calibration and configuration values are stored in the microcontroller EEPROM. These values are read and used each time the probe is switched on. When the command 0 × 78 is sent to the probe, the reading of the temperature value with the ADC and stored on the 0 × 20 and 0 × 30 addresses of the EEPROM is executed. If the compensation mode is activated, the frequency value is adjusted before being sent. To confirm the update of the calibration value, this value is sent to the acquisition module. To change the probe’s address, the 0 × 61 command is used and it necessary to reset the probe for the changes to take place.

The circuit used to measure the temperature using a thermistor uses 0.25 mW in a 25 °C environment. To have a maximum precision reading, it is necessary to keep the probe off when idle, as the continuous energy usage by the thermistor can interfere with read value.

**Figure 27:**
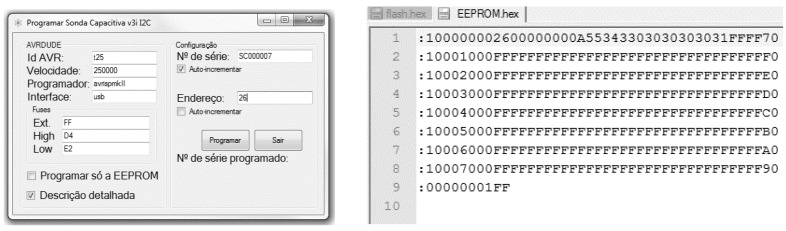
Programming application for the capacitive probe.

To have a simple configuration process for the probes using a computer, an application, using an USB-UART converter connected to a microcontroller, programed to interface between the UART port of the converter and the probe’s I2C port was developed. This application is very useful, considering that the communication protocol with the probe was designed and optimized for communication between electronic devices, making it hard to use manually by humans. To further automate the programming and configuration of the probes in batches, an application that generates an Intel HEX file [39] and programs the EEPROM was developed, as shown in Figure 27.

After compilation, the firmware in Intel HEX format is stored in a predefined file folder and the EEPROM is generated, based on the configuration data. To finish the process, the AVRDUDE application [40] with the parameters previously defined in the programming application is used, as shown in Figure 27, which programs the firmware and the generated EEPROM, and the microcontroller fuses [41], concluding the process.

## 4. Discussion and Conclusions

The main objective of this work was to create a simple and inexpensive system to measure the level of FOG in the grease boxes of industrial kitchens. The system was validated in real production facilities, thanks to a company dedicated to collecting and recycling fats and oils, which provided access to the industrial facilities.

From the several probes and sensors built, the best results were from the capacitive probes, as exposed in the previous section “Level Detection: Implementation and Results”. The capacitive probes were used for a complete system that was implemented and tested in production environment. A communication protocol was specified in order to facilitate the exchange of messages and the GSM network and SMS messages were used. After the probe development, the software needed to support the alert system, including the probe’s firmware, acquisition modules firmware and the data reception software, was also developed. The received data were saved in a database, in order to facilitate the integration with a third party management system.

The hardware and software components of the detection system were carefully chosen and integrated in order to minimize the costs of a future industrial production and, in the current market context, it represents a competitive solution. Due to the generalization of the detection with capacitive probes [21] and the low production cost, this probe may be a good option for other usages, such as water level monitoring in reservoirs, or even solid products, such as cereals silos.

## 5. Other Application and Future Work

As previously stated, these probes are good candidates for other usages as standalone elements or as a group of elements forming a single device. This scenario was studied and several tests were conducted to confirm it as a future work possibility.

**Figure 28:**
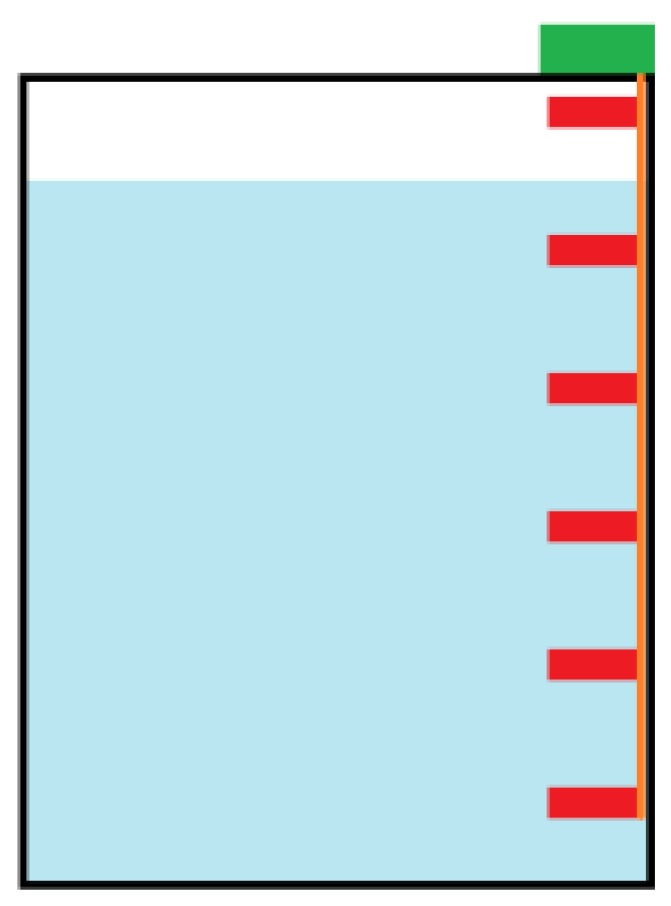
Several probes (in red) inside a container filled with water (in blue) are connected to a bus (in orange) and the acquisition module (in green).

Several probes can be connected to a unique acquisition module using the I2C protocol, which allows the connection of several probes to the same communication backplane, as shown in Figure 28. The number of probes is limited by the number of available addresses [37] and the 400-pF limit of the capacitance in the clock and data lines [36]. The capacitance limit problem can be overcome by using I2C repeaters [42], which set the limit at the 112 available addresses.

**Figure 29:**
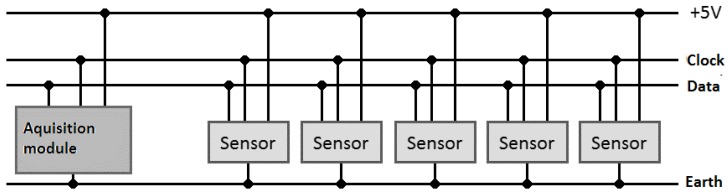
Communication of a master I2C with several slave sensors in the same bus.

The probes in Figure 28 can be connected (Figure 29) so a continuous value is obtained with a maximum resolution proportional to the number of probes used.

To demonstrate the probe’s ability to differentiate different surrounding materials, using the probe’s frequency, several tests were done in which the probe was installed in a glass container and surrounded by different substances. Five readings were recorded for each substance. Table 5 shows the average of those readings.

The earth samples were collected from the same place, before and after an irrigation cycle. The results show a clear distinction between the dry and wet earth.

## Figures and Tables

**Table 1:** The probe’s readings at different temperatures.

Temperature (°C)	ADC	Frequency (Hz)
10	704	87,303
11	696	87,026
12	684	86,873
13	671	86,545
14	655	85,988
15	639	85,670
17	624	85,288
18	608	84,937
21	560	83,427
24	526	82,833

**Table 2:** Compensated results from readings in Table 1.

Temperature (°C)	ADC	Frequency (Hz)	Compensated (Hz)
10	704	87,303	84,903
11	696	87,026	84,826
12	684	86,873	84,973
13	671	86,545	84,970
14	655	85,988	84,813
15	639	85,670	84,895
17	624	85,288	84,888
18	608	84,937	84,937
21	560	83,427	84,627
24	526	82,833	84,883

**Table 3:** Command to configure and operate the probe.

Command (byte)	Operation Description	Parameters (sent bytes)	Reply (received bytes)
0 × 61	Aquire sample	0	6
0 × 65	Change I2C address	1	1
0 × 63	Define CAL	2	2
0 × 6B	Define k	1	1
0 × 6C	Read configuration	0	4
0 × 74	Read temperature	0	2
0 × 78	Calibrate	0	2
0 × 43	Turn compensation on/off	1	1
0 × 73	Read serial number	0	8

**Table 4:** Bytes received after a sample reading request.

Byte	1	2	3	4	5	6
Value in Figure 26	0 × 00	0 × 02	0 × F4	0 × 01	0 × F2	0 × 16
	Frequency (MSB)	Frequency	Frequency (LSB)	ADC	ADC	Check

**Table 5:** Readings average with the probe in different substances.

Substance	Frequency (Hz)	Temperature (°C)
Water	4450	24
Air	10,617	27
Rice	8310	23
Corn	6857	23
Moist sand	4510	23
Dry earth	10,555	25
Wet earth	5917	20
